# Identification of milling status based on vibration signals using artificial intelligence in robot-assisted cervical laminectomy

**DOI:** 10.1186/s40001-023-01154-y

**Published:** 2023-06-29

**Authors:** Rui Wang, He Bai, Guangming Xia, Jiaming Zhou, Yu Dai, Yuan Xue

**Affiliations:** 1 Key Laboratory of Spine and Spinal Cord, Department of Orthopedic Surgery, Tianjin Medical University General Hospital, Tianjin, 300052 China; 2 Tianjin Key Laboratory of Intelligent Robotics, Institute of Robotics and Automatic Information System, College of Artificial Intelligence, Nankai University, 94 Weijin Road, Nankai District, Tianjin, 300071 China

**Keywords:** Cervical laminectomy, Vibration signals, Fast Fourier transform, Artificial intelligence, K-nearest neighbors

## Abstract

**Background:**

With advances in science and technology, the application of artificial intelligence in medicine has significantly progressed. The purpose of this study is to explore whether the k-nearest neighbors (KNN) machine learning method can identify three milling states based on vibration signals: cancellous bone (CCB), ventral cortical bone (VCB), and penetration (PT) in robot-assisted cervical laminectomy.

**Methods:**

Cervical laminectomies were performed on the cervical segments of eight pigs using a robot. First, the bilateral dorsal cortical bone and part of the CCB were milled with a 5 mm blade and then the bilateral laminae were milled to penetration with a 2 mm blade. During the milling process using the 2 mm blade, the vibration signals were collected by the acceleration sensor, and the harmonic components were extracted using fast Fourier transform. The feature vectors were constructed with vibration signal amplitudes of 0.5, 1.0, and 1.5 kHz and the KNN was then trained by the features vector to predict the milling states.

**Results:**

The amplitudes of the vibration signals between VCB and PT were statistically different at 0.5, 1.0, and 1.5 kHz (*P* < 0.05), and the amplitudes of the vibration signals between CCB and VCB were significantly different at 0.5 and 1.5 kHz (*P* < 0.05). The KNN recognition success rates for the CCB, VCB, and PT were 92%, 98%, and 100%, respectively. A total of 6% and 2% of the CCB cases were identified as VCB and PT, respectively; 2% of VCB cases were identified as PT.

**Conclusions:**

The KNN can distinguish different milling states of a high-speed bur in robot-assisted cervical laminectomy based on vibration signals. This method is feasible for improving the safety of posterior cervical decompression surgery.

## Background

Cervical spondylotic myelopathy (CSM), the most common cause of spinal dysfunction, is caused by chronic segmental compression of the spinal cord due to spondylotic change [1, 2]. More than 80% of people experience cervical disc degeneration observable using magnetic resonance imaging by the age of 50 years [3, 4]. If conservative treatment fails or neurological signs and symptoms worsen, surgery is the only effective procedure for treating patients [5]. According to the different pathological conditions of CSM, surgical methods include anterior surgery, posterior surgery, and combined anterior and posterior surgery [5–9].

In cervical laminectomy, milling the bone with a high-speed bur is one of the most common operations [10, 11]. The milling process with a high-speed bur is performed in a narrow space, which makes the spinal cord, nerve root, blood vessels, and other important structures vulnerable during the operation. If the bur slips and penetrates the spinal canal, it can lead to serious surgical complications, including spinal cord injury, dural sac injury, cerebrospinal fluid leaks, nerve root injury, and peripheral vascular injury [10–12]. Therefore, monitoring the milling state of the high-speed bur is important to ensure safety during cervical laminectomy.

To avoid these risks, multiple sensors have been used to monitor the milling states, such as the feedback of torque and force changes [13, 14], image signal feedback [15], electrical impedance [16, 17], and current and voltage sensors [18]. Dai et al. successfully identified the critical state before lamina penetration by analyzing the vibration and sound pressure signals generated during the process of milling a pig spine [10, 19]. Although some surgical robots have been used in orthopedic surgery [20, 21], spinal surgery robots, such as Mazor SpineAssist, Mazor Renaissance, and Rosa Spine among others, are mainly limited to spinal fusion and internal fixation; these robots set the pedicle screw placement trajectory with the help of a preoperative imaging system and rely on the surgeon to complete the screw placement process [22–24]. Moreover, random events such as the patient's intraoperative movement and cannula sliding result in a catastrophic error in the screw positioning offset [22, 25]. Owing to the complexity of the spine structure, the high risk of spinal surgery, and the long learning curve of surgery, the development of automatic spine milling surgery robots is constrained.

A high-speed bur is an exciting source of vibration signals, and because the ventral cortical bone (VCB) has a higher density than the cancellous bone (CCB), the vibration signal during the milling of the VCB is different from that during the milling of the CCB, which is a parameter that can provide useful information about the relative position between the high-speed bur blade and bone [26, 27]. Spinal surgeons with skilled surgical technology can judge the milling states of a high-speed bur by the vibration feedback produced during the surgery to avoid damage to the vital anatomy [28]; at present, all surgical robots lack vibration feedback. We performed this study to collect and analyze vibration signals during surgery. Then, a fast Fourier transform (FFT) was used to extract the harmonic amplitude of the vibration signals to construct feature vectors (FVs), which were used to train the k-nearest neighbors (KNN) algorithm to predict the milling states in robot-assisted cervical laminectomy.

## Methods

### Specimen preparation

Because the porcine spine is anatomically similar to the human spine [29], it was selected for the ex vivo experiment. Four fresh cervical spinal specimens were obtained from four-month-old mini pigs (weight range, 23–30 kg; four females and four males). The spinous processes and all nonessential soft tissues were dissected and carefully excised; only the facet joint capsules and ligamentous structures were preserved, and the bone was kept intact. The surgical area of the cervical specimen was fixed on the operating table using chucking fixtures. During surgery, the specimens were sprayed with physiological saline to keep the sites cool.

### Apparatuses for vibration measuring system

**Fig.1:**
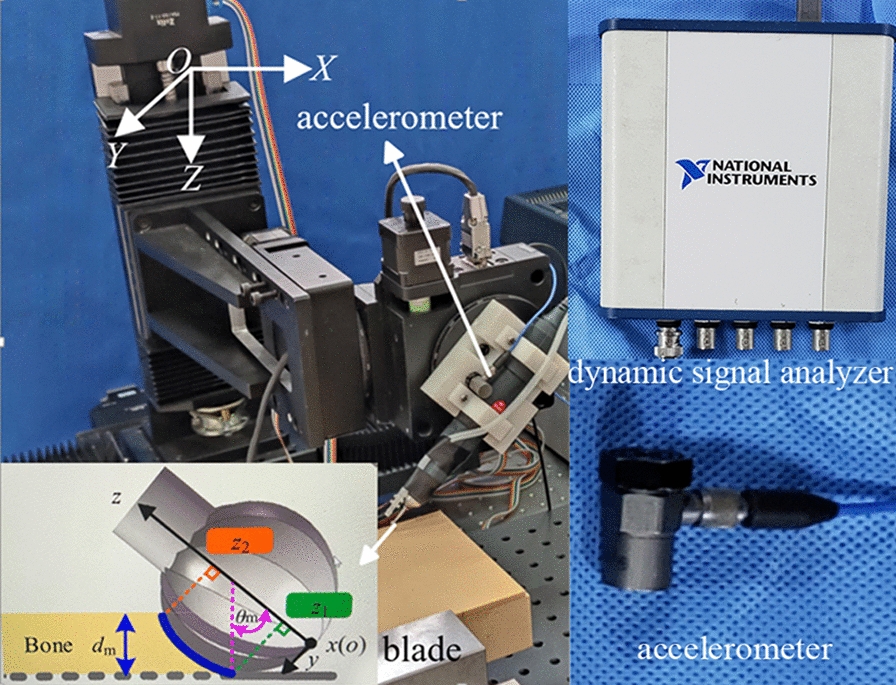
Three degrees of freedom stepping motor. For each motion axis, the stepping motor produces linear movement. The end of the robot arm is equipped with an accelerometer

The vibration signal measurement system can be divided into two parts: the power and vibration signal acquisition parts. The power part comprises a laboratory-built stepping motor equipped with a GD676 high-speed bur (B. Braun vet care GmbH, Tuttlingen, Germany); the rotating speed is set to 30,000 rpm and melon bur blades with outer diameters of 5 and 2 mm are installed. The end of the robot arm is equipped with a vibration sensor and a physiological saline irrigation pipe. As shown in Fig. 1, the stepping motor (OMAP-l137dsp; Texas Instruments, Dallas, TX, USA) can control the cutting position, depth, and speed in the three translation axes. In the vibration signal acquisition part, the vibration signals are collected and recorded using a uniaxial accelerometer (PCB Piezoelectric Electronics Company, New York, NY, USA) and a USB-4431 dynamic signal analyzer (National Instruments, Austin, TX, USA). The frequency response range of the accelerometer is 0.5–200,000 Hz, its sensitivity is 100 mV/g and its measurement range is ± 50 g, In the study, the frequency response was set at 12.8 kHz. The signal analyzer provides 24-bit resolution and a maximum sampling frequency of 102.4 kHz.

### Surgical method

**Fig. 2:**
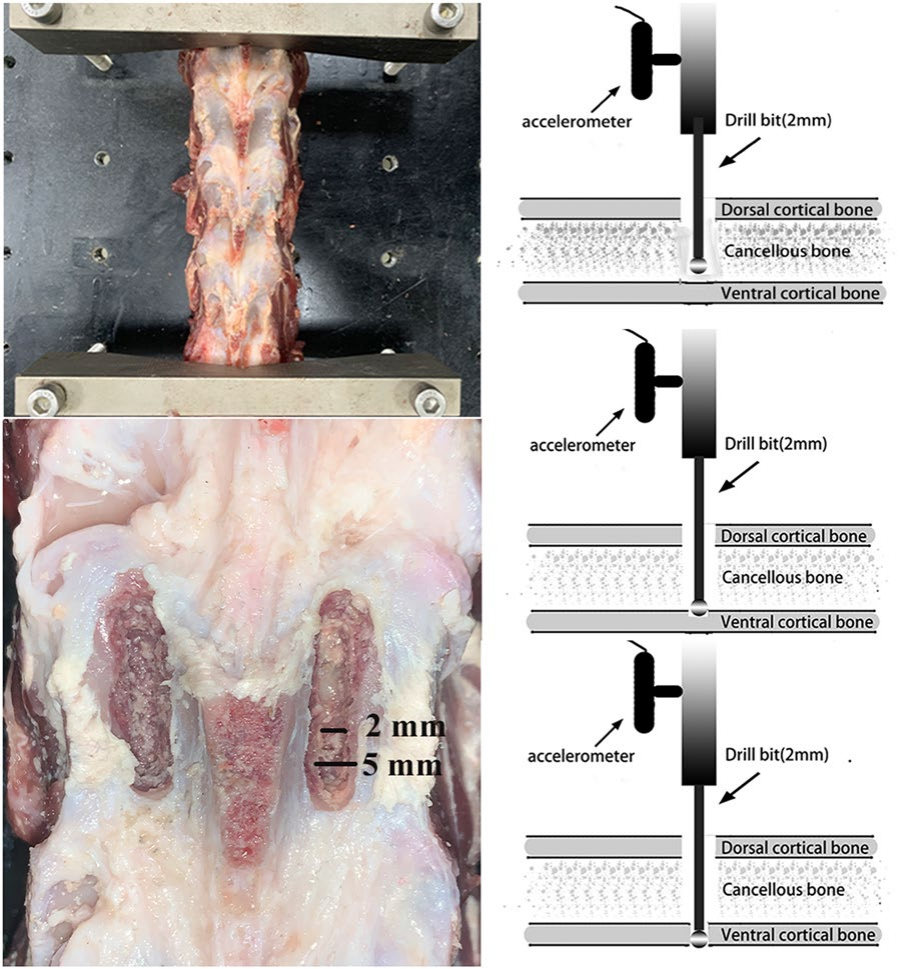
Milling methods of the high-speed bur

First, the 5 mm melon bur blade was used to mill the dorsal cortical bone along the longitudinal axis of the lamina to form bone grooves on both sides. Second, the 2 mm melon bur blade was used to mill the CCB until penetration (PT) on both sides (Fig. 2). At this stage, laminae were completely removed. The bur blade was cooled using physiological saline during surgery. In the milling process, the mechanical arm automatically feeds as per the set program. The robot first feeds along the Z-axis direction to depth 1 mm, and then it moves at a constant speed of 0.5 mm/s in the Y-axis direction to execute high-speed bur milling. The experiment was conducted in accordance with animal care guidelines and approved by the Animal Ethics Committee of the University.

### Analysis of bone milling process and design of the experiments

**Fig. 3:**
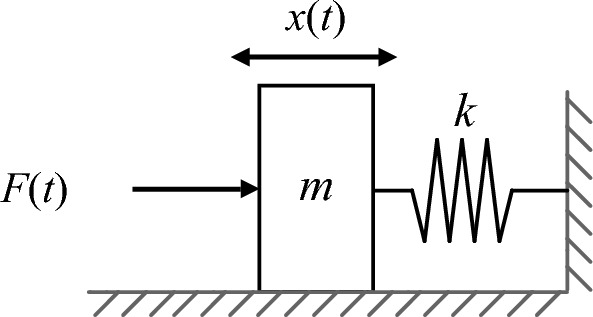
Dynamics model of the milling force *F(t)*

The cervical lamina at the milling site is connected to the vertebral body, muscles, ligaments, and intervertebral discs. The mechanical-physical model of the milling site is equivalent to a single-degree-of-freedom spring-mass model in the feed direction (Fig. 3). The forced vibration Eq. (1) is as follows:

1 $$m\ddot{x}\left(t\right)+kx\left(t\right)=F\left(t\right)$$

where *m* and *k* denote the equivalent mass and stiffness of the musculoskeletal system, respectively. *x(t)* is the displacement of *m* from the equilibrium position, $\ddot{X}$*(t)* is the second derivative of the displacement, and the milling force *F(t)* is the periodic harmonic force. Only the dynamic of the bone being drilled is investigated,

2 $$F(t)= {F}_{o} +\sum_{i=1}^{L}{F}_{p}\mathrm{sin}(2\mathrm{\pi i}{f}_{r}\mathrm{t}+{\mathrm{\varphi }}_{i})$$

The steady-state solution for (1) and (2) is Eq. (3):

3 $$\ddot{\text{x}}\left(t\right)\text{=}{\sum }_{\text{i=1}}^{\text{L}}\frac{{4}{{\pi }^{2}i}^{2}{f}_{r}^{2}{F}_{p}}{k-4{\pi }^{2}m{i}^{2}{f}_{r}^{2}}\mathit{sin}\left(2\pi i{f}_{r}t+{\varphi }_{i}\right)$$

where *f*_*r*_ is the rotation frequency of the spindle of the high-speed bur, *F*_*p*_ denotes the amplitude of the milling harmonic force, ${F}_{o}$ denotes the constant force, *i* denotes the *i*th harmonic, and *φ*_*i*_ is the initial phase angle of the *i*^th^ milling harmonic.

The periodic milling force *F(t)* is related to the bone removal volume *V* and bone mineral density *ρ*, where γ is the coefficient of bur blade structure and bur rotation speed (related to blade structure and bur rotation speed), *k*_*γ*_*、k* $\rho$ and *k*_*v*_ are the exponential coefficient of *γ* $, \rho$ and *V* respectively. This relationship is expressed in Eq. (4):

4 $${F}_{p}={\gamma }^{{k}_{\gamma }}{\rho }^{{k}_{\rho }}{V}^{{k}_{V}}$$

**Fig. 4:**
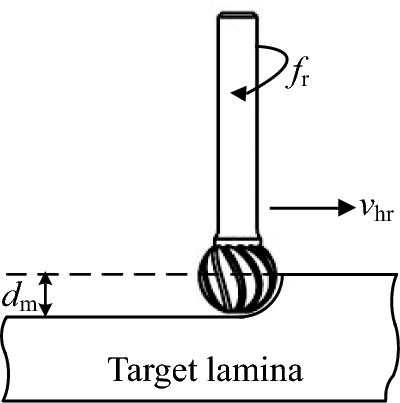
Milling states of the high-speed bur blade in the single degree of freedom direction

The milling states of the high-speed bur blade in the horizontal direction (Fig. 4) were analyzed. Because the bur blade is a melon blade and the cutting depth d_m_ did not exceed the radius r of the bur blade, the cutting contact area *A*_*hz*_ was proportional to the cutting depth *d*_*m*_*,* as shown in Eq. (5).

5 $${A}_{hz=}\pi R{d}_{m}$$

At the feed rate $\upnu$_*hz*_, there is instantaneous bone removal volume *V*_*hz*_, which is related to milling contact area *A*_*hz*_ and milling speed ${v}_{hz}$, as shown in Eq. (6):

6 $${V}_{hz}={A}_{hz}{v}_{hz}$$

On solving Eqs. (3–6), we obtain Eq. (7):

7 $$\ddot{\text{x}}\left(t\right)\text{=}{\sum }_{\text{i=1}}^{\text{L}}{\gamma }^{{k}_{\gamma }}{\rho }^{{k}_{\rho }}\left(\pi R{d}_{m}{v}_{hz}\right)\frac{{4}{{\pi }^{2}i}^{2}{f}_{r}^{2}\mathit{sin}\left(2\pi i{f}_{r}t+{\varphi }_{i}\right)}{k-4{\pi }^{2}m{i}^{2}{f}_{r}^{2}}$$

Given that $\upzeta =\frac{{4}{{\pi }^{2}i}^{2}{f}_{r}^{2}\mathit{sin}\left(2\pi i{f}_{r}t+{\varphi }_{i}\right)}{k-4{\pi }^{2}m{i}^{2}{f}_{r}^{2}}$, we obtain Eq. (8):

8 $$\ddot{\text{x}}\left(t\right)\text{=}{\sum }_{\text{i=1}}^{\text{L}}{\gamma }^{{k}_{\gamma }}{\rho }^{{k}_{\rho }}\left(\pi R{d}_{m}{v}_{hz}\right)\zeta$$

According to Eq. (8), the influence on the vibration signals harmonic amplitudes of high-speed bur are mainly the structure of the bur blade and the rotation speed of the bur (*γ)*, milling depth (*d*_*m*_), milling speed $(\upnu$_*hz*_*)* and bone density (*ρ),* In the in vitro experiment, we controlled the diameter of the bur blade to be 2 mm, the depth to be 1 mm, and the speed to be 0.5 mm/s, and we uniformly standardized and fixed the spinal tissues to be ground on the operating table (Daeil Systems, Yongin, Republic of Korea), which reduced the influence of $\zeta$. By controlling the variables, the bone density (*ρ*) became the main factor affecting the vibration signals in the experiment.

## Results

**Fig. 5:**
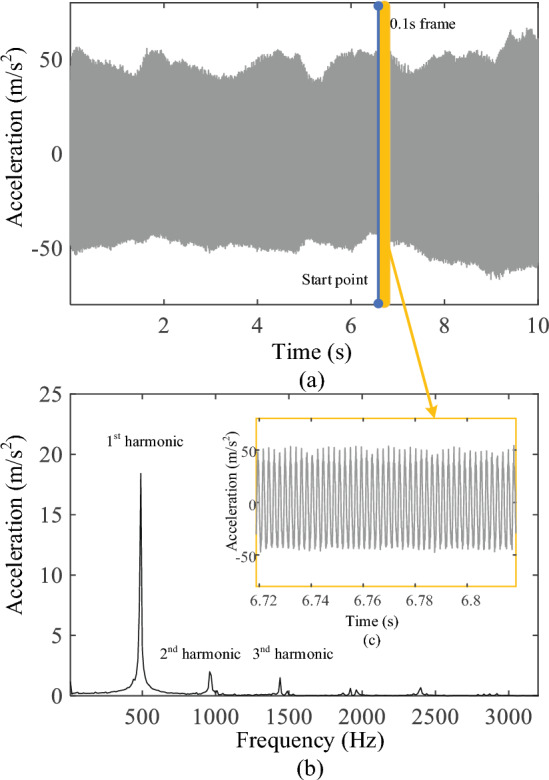
Vibration signals during milling cancellous bone. **a** Raw 10 s milling vibration signals; **b** FFT spectrum analysis of 0.1 s vibration signals; **c** 0.1 s frame of raw vibration signal

**Fig. 6:**
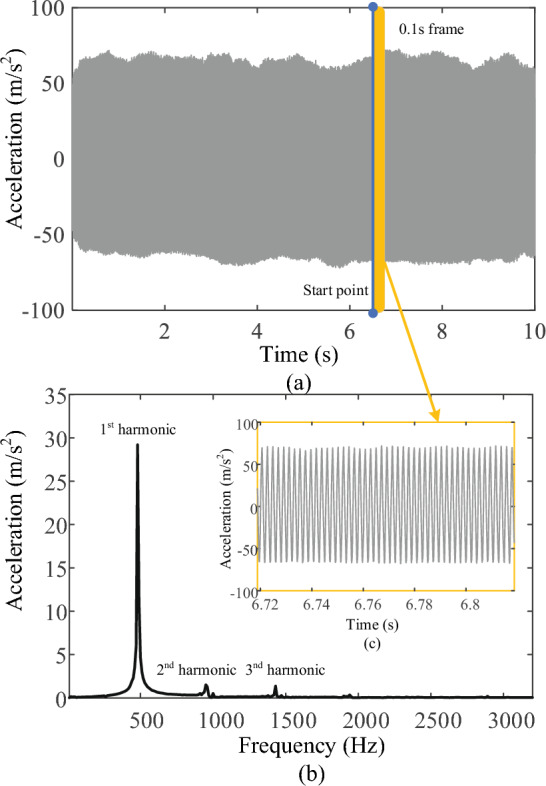
Vibration signals during milling ventral cortical bone. **a** Raw 10 s milling vibration signals; **b** FFT spectrum analysis of 0.1 s vibration signals; **c** 0.1 s frame of raw vibration signal

In the process of milling the lamina, continuous 10 s vibration signals were collected in each state, and all the collected data were divided into 0.1 s/frame, with each frame containing 1280 vibration signal data points. FFT was used to process the vibration signals and extract the amplitude of the first three harmonic signals (0.5, 1.0, and 1.5 kHz) using the MATLAB (2022a; MathWorks, Natick, MA, USA) software on a personal computer, as shows in Table 1. The Cartesian coordinate system was built as shown in Figs. 5, 6and7. An independent sample t-test was performed for statistical analysis. *P* < 0.05 was considered significant difference. In the frequency band at approximately 0.5 kHz, there was a significant difference in the vibration signal amplitudes between the CCB and VCB (*P* < 0.001) and between the VCB and PT (*P* < 0.001). The amplitudes of the vibration signal at a frequency of 1.0 kHz showed significant differences between VCB and PT (*P* < 0.05); no significant difference was observed between VCB and CCB. In the frequency band at approximately 1.5 kHz, there was a significant difference between the VCB and CCB (*P* < 0.01) and between the VCB and PT (*P* < 0.05), as shown in Fig. 8.

**Table 1:** The amplitude: mean ± standard deviation for 0.5, 1.0, and 1.5 kHz in each milling state

(Amplitude: m/s^2^)	0.5 kHz	1.0 kHz	1.5 kHz
CCB	18.2295 ± 3.3964	6.2323 ± 2.8972	2.8369 ± 0.7864
VCB	32.4975 ± 6.7584	6.4679 ± 3.3727	4.8487 ± 1.4698
PT	13.4627 ± 0.7410	3.7030 ± 0.4303	3.3998 ± 1.0425

*CCB* cancellous bone, *VCB* ventral cortical bone, *PT* penetration

**Fig. 7:**
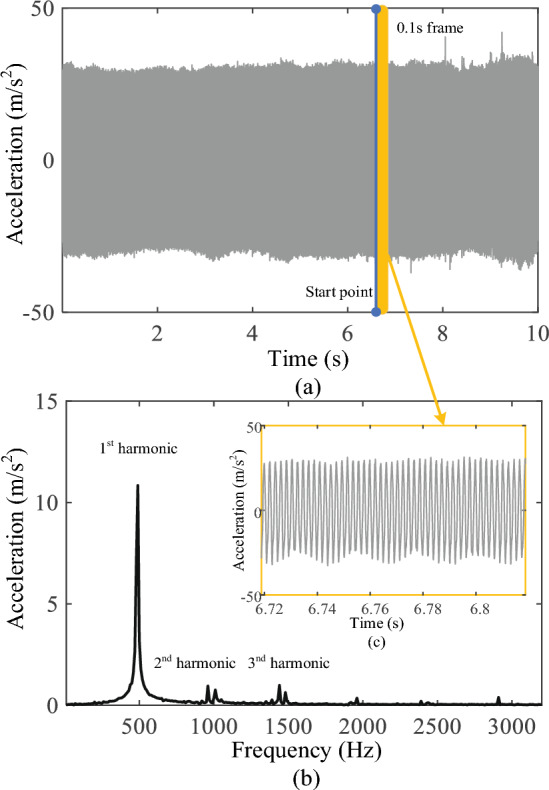
Vibration signals of the penetration. **a** Raw 10 s milling vibration signals; **b** FFT spectrum analysis of 0.1 s vibration signals; **c** 0.1 s frame of raw vibration signal

**Fig. 8:**
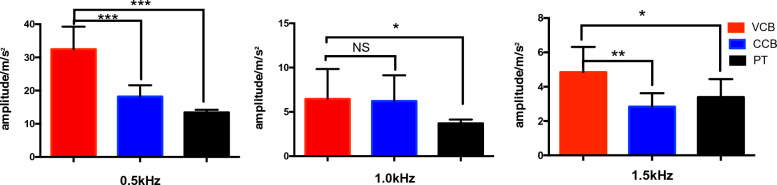
Comparison of vibration signals amplitudes at different milling states and different frequencies. NS: There was no statistical significance (*P* > 0.05); *: was statistically significant (*P* < 0.05); **: was statistically significant (*P* < 0.01); and ***: was statistically significant (*P* < 0.001)

**Fig. 9:**
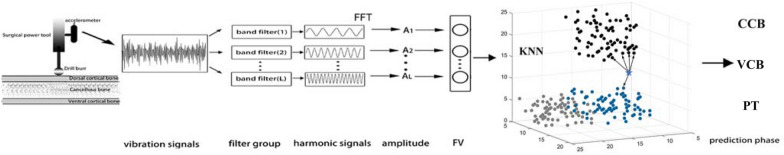
Dynamics model of vibration signal processing and milling state identification

The vibration signal amplitudes corresponding to the frequencies were considered features, and the milling states were used as labels. The FV [milling states; feature 1; feature 2; feature 3…] was constructed to train the KNN to automatically identify the three milling states (CCB, VCB, and PT). In the experiment, 200 sets of vibration signals were collected for each state to construct the FV. Seventy percent of the samples were randomly selected from the three milling state samples as training samples and the remaining groups were used as test samples to identify the milling states of the high-speed bur. The processes of vibration signal processing and milling state recognition are illustrated in Fig. 9.

**Fig. 10:**
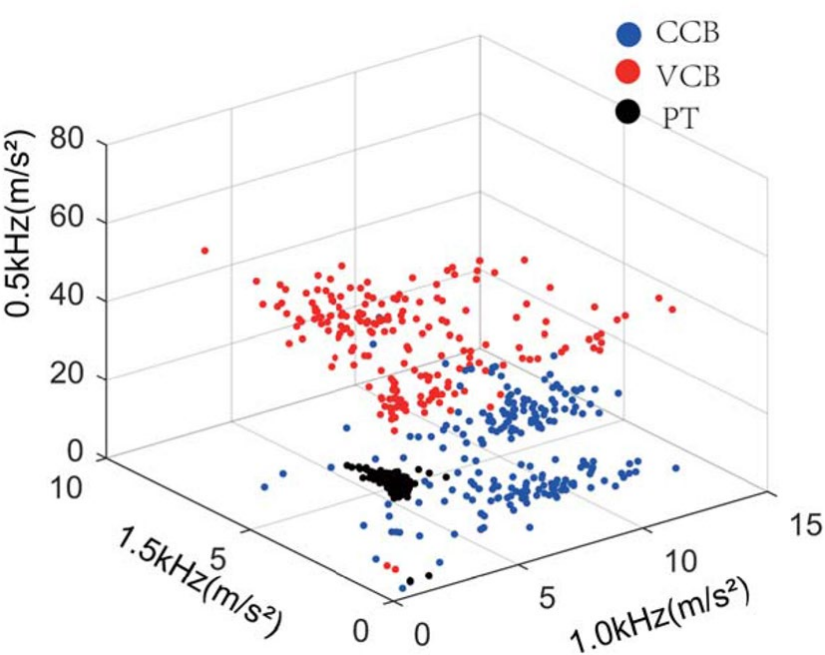
Three-dimensional feature space (KNN) under different milling states

The different frequency amplitudes of the vibration signals were extracted using FFT as the X-, Y-, and Z-axes to construct a three-dimensional feature space. A scatter diagram of the 600 groups in different milling states is shown in Fig. 10. The FV selected in this coordinate system better reflected the characteristics of the different milling states. Table 2 shows the recognition accuracy of KNN in the feature space; the recognition rates of the three milling states were all greater than 92%. The recognition rate for PT was 100%; 6% and 2% of CCB were identified as VCB and PT, respectively, and 2% of VCB was identified as PT.

**Table 2:** Confusion matrix of milling state recognition based on KNN (k = 5)

Recognition rate (%)	CCB	VCB	PT	Total
CCB	92	6	2	–
VCB	0	98	2	–
PT	0	0	100	–
Total	–	–	–	98

*CCB* cancellous bone, *VCB* ventral cortical bone, *PT* penetration

## Discussion

CSM results from degenerative cervical changes. The neurological symptoms of myelopathy are caused by spinal canal stenosis leading to spinal cord compression, which is the most common cause of spinal cord dysfunction. More than 112,400 cervical spine operations are performed in the United States every year [30]. A prospective study showed that the incidence of complications in CSM surgery is high (17% in 2013) [31], especially in elderly patients aged over 74 years [32]. Therefore, improving spinal surgery safety is a major issue that must be addressed. Surgical-assisted robotic surgery has been implemented because of its low trauma, high precision, and strong stability, demonstrating success in surgical operations for colectomy, radical prostatectomy, cholecystectomy, and myectomy, among others [33–37]. For example, the Da Vinci surgical robot has been used in general surgery and urology, and shows superior visualization and magnification compared to traditional laparoscopy [38–40]. However, because of the complex structure of the spine, the narrow surgery operation space, and the important anatomical structures around the spine, the automatic milling spine surgery robot has not been clinically applied. SpineAssist (Mazor Robotics Ltd., Caesarea, Israel) was the first spinal robot approved by the United States Food and Drug Administration in 2004 [41–43] and is limited to intraoperative navigation and stereotactic positioning; it also requires patient the preoperative CT scan [25].

To improve the safety of the high-speed bur in cervical laminectomy, this study explores a monitoring method for the milling states based on vibration feedback. According to the derivation of Eq. (8), the density of vertebral lamina, milling depth, milling speed, the structure and rotating speed of bur blade affect the vibration signals. In this experiment, we control the bur blade to 2 mm, the bur rotation to 3000 rpm, the milling depth to 1 mm, and the milling speed to 0.5 mm/s; the cervical vertebrae tissues are also fixed on the operating table (Daeil systems, Yongin, Republic of Korea) to reduce the influence of the environment. Most importantly, the density of tissue is a unique variable that affects the vibration signal. Based on our previous research, the harmonic frequency of a vibration signal is an integral multiple of the spindle frequency of the high-speed bur [10, 11, 27]. Different milling states can be distinguished by comparing their harmonic amplitudes.

The vibration signal characteristics of the milling states were extracted using FFT. Through the independent sample t-test analysis, as for the amplitudes of the vibration, it is concluded that at the frequencies of 0.5 and 1.5 kHz, there were a significant difference between the CCB and VCB, and between the VCB and PT (*P* < 0.05). At a frequency of 1 kHz, there was a significant difference between the VCB and PT (*P* < 0.05), but there was no significant difference between the VCB and CCB. The amplitude of each frequency for the vibration signals was used to construct the FV. The milling states were then identified using KNN. As shown in Table 2, the recognition rates of the three milling states were greater than 92%, indicating that the identification characteristics of the vibration signals could distinguish the three working states well. The recognition rate of the penetrating state was 100%. Although 6% and 2% of CCB were identified as VCB and PT, respectively, and 2% of VCB were identified as PT, all relatively dangerous states were identified as more hazardous states, which is conducive to safe lamina decompression. In conclusion, vibration signals can be used to identify the milling state of a high-speed bur in robot-assisted cervical laminectomy.

The sampling and analysis time of 0.1 s is sufficient to realize real-time state identification. A physiological saline-washing bur blade was used to reduce the high temperature of the drill blade and protect the structure during operation. The advantages of this method include non-contact vibration measurement feedback, unaffected intraoperative operation, fast response, simple process, high accuracy, less burden on surgeons, and less intraoperative fluoroscopy, which will help novice surgeons better control high-speed burs, improve operation safety, and reduce radiation damage to doctors and patients.

This study has some limitations. The in vitro experiment did not exhibit the complete damping and elastic modulus required for the in vivo experiment. All samples were vertebrae of normal animals, and pathological structures were not included in the experimental models. Owing to experimental limitations, soft tissues, such as ligaments, can easily cause winding during the milling process, which will affect the collection of vibration signals. Therefore, the vertebral lamina was stripped of soft tissue. In the future, we will conduct further in vivo experiments, increase the physical quantities of damping and elasticity, analyze the impact of rotation speed, milling depth, and milling speed of the bur on vibration signals, and integrate the vibration signal analysis and feedback system into the chip to realize real-time monitoring and control of milling states.

## Conclusion

In robot-assisted cervical laminectomy, artificial intelligence using KNN can distinguish different milling states of a high-speed bur based on vibration signals. This provides a monitoring method to improve the safety of surgery. In the future, a multi-vibration sensor array will be applied in robot-assisted laminectomy to ensure the accuracy of milling state recognition under complex conditions.

## Data Availability

The data used during the study are available from the corresponding author on reasonable request.

## Abbreviations

CSM: Cervical spondylotic myelopathy
KNN: K-nearest neighbors
FFT: Fast Fourier transform
CCB: Cancellous bone
VCB: Ventral cortical bone
PT: Penetration
